# Examining the Effects of the Nitrogen Environment on Growth and N_2_-Fixation of Endophytic *Herbaspirillum seropedicae* in Maize Seedlings by Applying ^11^C Radiotracing

**DOI:** 10.3390/microorganisms9081582

**Published:** 2021-07-25

**Authors:** Spenser Waller, Stacy L. Wilder, Michael J. Schueller, Alexandra B. Housh, Stephanie Scott, Mary Benoit, Avery Powell, Garren Powell, Richard A. Ferrieri

**Affiliations:** 1 Missouri Research Reactor Center, University of Missouri, Columbia, MO 65211, USA; sgwxhv@mail.missouri.edu (S.W.); wildersl@missouri.edu (S.L.W.); schuellerm@missouri.edu (M.J.S.); afbkhn@mail.missouri.edu (A.B.H.); srstt9@mail.missouri.edu (S.S.); bvbenoit@mail.missouri.edu (M.B.); apgg4@mail.missouri.edu (A.P.); garren.powell@mail.missouri.edu (G.P.); 2 School of Natural Resources, University of Missouri, Columbia, MO 65211, USA; 3 Chemistry Department, University of Missouri, Columbia, MO 65211, USA; 4 Biochemistry Department, University of Missouri, Columbia, MO 65211, USA; 5 Division of Plant Sciences, Interdisciplinary Plant Group, University of Missouri, Columbia, MO 65211, USA

**Keywords:** *Herbaspirillum seropedicae*, green fluorescence reporting, endophytic rhizobacteria, maize roots, carbon-11, plant-borne carbon

## Abstract

*Herbaspirillum seropedicae,* as an endophyte and prolific root colonizer of numerous cereal crops, occupies an important ecological niche in agriculture because of its ability to promote plant growth and potentially improve crop yield. More importantly, there exists the untapped potential to harness its ability, as a diazotroph, to fix atmospheric N_2_ as an alternative nitrogen resource to synthetic fertilizers. While mechanisms for plant growth promotion remain controversial, especially in cereal crops, one irrefutable fact is these microorganisms rely heavily on plant-borne carbon as their main energy source in support of their own growth and biological functions. Biological nitrogen fixation (BNF), a microbial function that is reliant on nitrogenase enzyme activity, is extremely sensitive to the localized nitrogen environment of the microorganism. However, whether internal root colonization can serve to shield the microorganisms and de-sensitize nitrogenase activity to changes in the soil nitrogen status remains unanswered. We used RAM10, a GFP-reporting strain of *H. seropedicae,* and administered radioactive ^11^CO_2_ tracer to intact 3-week-old maize leaves and followed ^11^C-photosynthates to sites within intact roots where actively fluorescing microbial colonies assimilated the tracer. We examined the influence of administering either 1 mM or 10 mM nitrate during plant growth on microbial demands for plant-borne ^11^C. Nitrogenase activity was also examined under the same growth conditions using the acetylene reduction assay. We found that plant growth under low nitrate resulted in higher nitrogenase activity as well as higher microbial demands for plant-borne carbon than plant growth under high nitrate. However, carbon availability was significantly diminished under low nitrate growth due to reduced host CO_2_ fixation and reduced allocation of carbon resources to the roots. This response of the host caused significant inhibition of microbial growth. In summary, internal root colonization did little to shield these endophytic microorganisms from the nitrogen environment.

## 1. Introduction

One of the most promising agro-ecological approaches that could enable a reduction in use of synthetic N fertilizer is the exploitation of N_2_-fixing (BNF) bacteria. A variety of BNF bacteria are commonly present in the plant rhizosphere that can establish close associations with roots, colonizing the roots either epiphytically or endophytically [1,2,3]. Indeed, plant growth promoting bacteria (PGPB) can reach dense concentrations (e.g., 10^8^/g) in roots without inducing a noticeable plant defense response [4,5,6]. Previous studies have shown that PGPB commonly impact root architecture and plant health, attributing these effects to such things as BNF, production of phytohormones, and enhancement of nutrient acquisition, which can protect hosts against pests and pathogens and build tolerances to extreme climatic conditions [7,8].

BNF relies on nitrogenase to catalyze the energy-demanding reduction of N_2_ to ammonia coupled with H_2_ production [9]. This enzyme is composed of two protein components: one composed of a MoFe protein, and the other a Fe protein. The action of nitrogenase is supplemented by ferredoxin acting as a reductant and Mg-ATP as an energy source. Under optimal growth conditions, nitrogenase requires 16 Mg-ATP molecules for the reduction of 8H^+^ + N_2_ → 2 NH_3_ + H_2_. However, more typically between 20 to 30 Mg-ATP molecules are consumed in the process to reduce a single molecule of N_2_ [9].

Because BNF comes at such a high energy cost to the microorganism, tight control over nitrogenase synthesis and its level of activity are important to the overall health of the microorganism. Past studies have shown that the *nif* genes responsible for regulating nitrogenase activity are typically repressed in the presence of extraneous nitrogen sources such as ammonium [10,11] and nitrate [12]. Typically, these N_2_-fixing organisms turn off nitrogenase activity in the presence of these extraneous N sources and turn it back on when these sources become exhausted through microbial metabolism.

Past studies mostly within *Azospirillum* spp. have also shown that besides genetic control over nitrogenase activity, the enzyme can be regulated at a metabolic level [13]. One of the interesting features for accomplishing this is through covalent modification of one of the subunits of dinitrogenase reductase by dinitrogenase reductase ADP-ribosyltransferase (DRaT) (for a review, see [14]). The system can be reactivated when the ammonium or nitrate source is exhausted or upon addition of excess ATP, or divalent metals such as Mg^2+^ or Mn^2+^ that bind ATP, or by the addition of dinitrogenase reductase activating glycohydrolase (DRaG), which can remove the inactivating group re-instating normal nitrogenase activity [15,16].

Like the well-studied *Azospirillum* spp., *Herbaspirillum* is a Gram-negative N_2_-fixing bacterium that has been isolated from the roots of many cereal crops including rice, maize, and sorghum [17]. Though a covalent switch controlling nitrogenase activity in this bacterium is not evident as in *Azospirillum* spp., the enzyme does exhibit significant inhibition in the presence of external nitrogen sources [11]. Because this bacterium is endophytic, choosing to colonize the inner compart of the plant root, and because it fixes N_2_ over a broader pH range (5.3 to 8.0) than *Azospirillum* spp., there is growing interest in its potential utility in agriculture where the root tissue may shield the bacterium from the soil environment.

Using a protocol recently established within our lab [18], we administered radioactive ^11^CO_2_ to track the incorporation of atmospheric carbon into plant carbohydrates with subsequent translocation of complex ^11^C-photosynthates to roots where they were assimilated by root colonizing *H. seropedicae* bacteria. Unlike stable isotope probing, our nuclear-based method was shown to be highly quantifiable, providing an absolute measurement of microbial assimilation of plant-borne ^11^C, which was used to examine changes in microbial “appetite” for carbon as a function of the surrounding environmental. Since our past studies showed that *H. seropedicae* strongly colonized the internal vascular core of maize roots rather than the outer root surface by 7-to-1 [18], we asked the fundamental question in the present work whether the host’s root tissue would desensitize the bacteria to changes in the external nitrogen environment.

## 2. Materials and Methods

### 2.1. Plant Growth and Root Inoculation with H. seropedicae Bacteria

Maize kernels from Elk Mound Seed Co. (Hybrid 8100, Elk Mound, WI, USA) were surface-sterilized in 5% bleach solution for 15 min, rinsed in sterile water, and dark germinated at room temperature for two days on sterilized paper towels wetted with sterile water in a Petri dish, and then allowed to grow for an additional 1–2 days before transferring them into plastic seed germination pouches (PhytoAB, Inc., San Jose, CA, USA) wetted with sterile Hoagland’s basal salt solution. Seedlings were allowed to grow for an additional week before transplanting them into 25 × 6 cm conical plastic pots filled with Turface™, a high-fired calcined clay matrix (Profile Product LLC., Buffalo Grove, IL, USA). Nutrient was introduced as Hoagland’s solution every 3 days, prepared either from Hoagland’s basal salt (1.63 g L^−1^: PhytoTechnology Laboratories, Shawnee Mission, KS, USA) for maintaining high nitrogen growth conditions at 10 mM nitrate or from a nutrient solution prepared using a commercially available no nitrogen Hoagland’s Basal salt (1.34 g L^−1^: Caisson Labs, Smithfield, UT, USA). This nutrient solution was enriched by the addition of KNO_3_ to achieve a low nitrogen growth condition at 1 mM nitrate. Growth conditions consisted of 12-h photoperiods, 500 μmol m^−2^ s^−1^ light intensity, and temperatures of 25 °C/20 °C (light/dark) with humidity at 70–80%. Plants were examined using ^11^C tracer to assess patterns for baseline ^11^CO_2_ fixation and root allocation of ^11^C-photosynthates in uninoculated plants at 3 weeks of age.

For plant stock that was inoculated with bacteria, we grew RAM10, a green fluorescence reporting strain of *H. seropedicae,* in liquid NFbHP-malate medium following published procedures [13]. The bacterial cultures were grown in 50 mL flasks in a shaking incubator set to 30 °C and 130 rpm until OD_600_ = 1.0 (optical density at 600 nm, corresponding to 10^8^ cells mL^−1^) was reached. Cultures were then washed with sterile water and diluted to a 1 mL suspended solution of approximately 10^6^ to 10^8^ colony-forming units (CFU mL^−1^). Inoculation of seedling roots involved adding 1 mL of inoculum to a Petri dish containing 10 germinated maize seedlings. The Petri dish was gently rocked in a shaking incubator for two hours before transferring inoculated seedlings into plastic germination pouches as described above. After an additional week of growth, seedlings were transplanted into 25 × 6 cm conical plastic pots filled with Turface™, and nutrient was administered using the same protocol as the uninoculated plant stock.

### 2.2. Production and Administration of Radioactive ^11^CO_2_

^11^CO_2_ (t_½_ 20.4 min) was produced on the GE PETtrace Cyclotron located at the Missouri Research Reactor Center using high-pressure, research-grade N_2_ gas target irradiated with a 16.4 MeV proton beam to generate ^11^C via the ^14^N(p,α)^11^C nuclear transformation [19,20]. The ^11^CO_2_ was trapped on molecular sieve, desorbed, and quickly released into an air stream at 200 mL min^−1^ as a discrete pulse for labeling a leaf affixed within a 5 × 10 cm lighted (560 μmol m^−2^ s^−1^) leaf cell to ensure a steady level of fixation. The load leaf affixed within the cell was pulse-fed ^11^CO_2_ for 1 min, then chased with normal air for the duration of exposure. A PIN diode radiation detector (Carroll Ramsey Associates, Berkeley, CA, USA) attached to the bottom of the leaf cell enabled continuous measurement of radioactivity levels within the cell during the initial pulse and in the minutes directly following to give information on ^11^CO_2_ fixation and leaf export of ^11^C-photosynthates [21].

Translocation of ^11^C-photosynthates to roots proceeded for one hour, after which plant tissues (shoots and roots) were collected and quantified for ^11^C-activity using a gamma-ray detector. Roots were first rinsed in de-ionized water to remove ^11^C-labeled root exudates. The Turface™ medium along with the root wash was counted for ^11^C-activity and combined with the root activity as a measure of root allocation.

### 2.3. Measuring Metabolic Partitioning of Plant Fixed ^11^C

In our examination of plant metabolism, leaves were exposed to ^11^CO_2_ for 20 min before tissues were excised, flash frozen in liquid nitrogen, ground to a fine powder, and extracted in methanol/water (60:40 v/v) via sonication (Branson, Bransonic 32; Sigma-Aldrich Corp., St. Louis, MO, USA) for 2 min at 100% amplitude in Eppendorf™ tubes. After centrifugation, the insoluble and soluble portions were separated and counted for ^11^C activity using a NaI gamma counter. The insoluble portion contained mostly cell wall polymers, starch, and other hydrophobic metabolites. The soluble portion contained small water-soluble compounds that were isolated using ion exchange chromatography. After rendering the extract slightly basic, we isolated the acidic metabolites from soluble leaf extract by their retention on an Accell QMA Plus Light Sep-Pak™ cartridge (Waters Corporation, Milford, MA, USA). This was connected in series to a SCX, strong cation exchange cartridge (Strata-XL-C, Phenomenex, Inc., Torrance, CA, USA), which retained basic metabolites. Upon rinsing both cartridges with deionized (DI) water, any neutral metabolites were eluted in the wash. Radioactivity associated with these metabolite fractions was then measured by NaI gamma counting of the ion exchange cartridges and the wash.

### 2.4. Autoradiography

After exposure to ^11^C tracer, plants were harvested, roots were laid out, and radiographic images were acquired by exposing phosphor plate films for 10 min on average. Phosphor plates were read using a Typhoon 9000 imager (Typhoon^TM^ FLA 9000, GE Healthcare, Piscataway, NJ, USA). Images were used qualitatively only for determining spatial patterning of ^11^C activity.

### 2.5. Fluorescence Imaging

A Typhoon 9000 imager was also used to perform low-resolution (100 μm) fluorescence imaging using blue laser excitation light (473 nm wavelength) and green light emission (ThorLabs, Inc., Newton, NJ, USA; filter no. MF525-39; 35 nm bandwidth centered at 525 nm) of the entire root mass. Roots were rinsed in deionized water before they were staged on a fluoroplate imaging cassette. This low-resolution image enabled identification of regions where there was a high level of microbial colonization by RAM10. After identifying regions of high microbial density, we cut 4-cm pieces from the total root mass, weighed them, and counted them for ^11^C activity before end-capping each piece in paraffin wax according to prior published procedures [18]. Sealed root sections were then subjected to 5-min sonication in a saline solution (1% NaCl) at 50% power (Branson Bransonic 32; Sigma-Aldrich Corp., St. Louis, MO, USA). The process had the effect of removing both surface-bound and internally colonized bacteria [18]. The saline solution was transferred to quartz optical quality cuvettes, imaged for their fluorescence signature using a Typhoon 9000 (quantified using ImageQuant TL 7.0 software), and counted for ^11^C activity.

### 2.6. Root-Associated Bacteria Growth Performance—Drop Plate Assay

Bacterial quantifications were performed concurrently with bacteria tracer studies. Plants were harvested from the growth media and rinsed in deionized water. Each experimental plant had two sections of root tissue harvested of approximately 3 cm in length to provide between 100 and 300 mg total sample for analysis. The combined fresh weight of tissue was recorded. The sample was shaken using a ball mill grinder with ball for approximately 5 min to macerate the tissue. Then, 1 mL of sterile 1% saline solution was added and vortexed with the sample. Five serial dilutions were performed: the first with 100 µL of the ground extract into 900 µL of 1% saline solution and each subsequent dilution, being 100 µL of the previous dilution into 900 µL of 1% saline solution. Each serial dilution was plated in triplicate by 10 μL drops onto agar plates fortified with malate growth media and incubated at 30 °C for 48–72 h before counting. ImageQuant software colony counting setting was used to quantify GFP exhibiting colonies measured on a Typhoon 9000 imager.

### 2.7. Liquid Culture Bacteria Growth Performance—Spectrophotometric Assay

RAM10 *Herbaspirillum seropedicae* were grown in liquid NFbHP-malate medium following the procedures describe above. The bacterial cultures were grown in 25 mL flasks in a shaking incubator set to 30 °C and 130 rpm using four levels of ammonium nutrient (as NH_4_Cl) as the added nitrogen source. Ammonium levels included 0.05, 0.25, 0.50, and 1.0 mM ammonium, where 0.5 mM was the typical amount of ammonium used for normal microbial growth [7]. The optical density of cultures was measured using an Evolution 201 UV–VIS spectrophotometer (Thermo Fisher Scientific, Inc., Waltham, MA, USA) set to measure absorption at 600 nm after 24 h of growth and 48 h of growth.

### 2.8. Microbial Nitrogenase Activity—Acetylene Reduction Assay

Acetylene reduction assays were completed to assess functionality of the nitrogenase enzyme under low-N and high-N plant growth conditions with microbial inoculation. Calcium carbide (1 g: Sigma-Aldrich Corp., St. Louis, MO, USA) was placed in a clean and dry 50 mL volume Falcon tube. The cap to the Falcon tube was modified to house a silicon rubber seal for connecting a 60 mL volume syringe filled with 30 mL DI water. Once water was introduced, acetylene was quickly produced and collected in a Tedler™ plastic gas collection bag (Sigma-Aldrich Corp., St. Louis, MO, USA).

Bacteria were grown in association with maize roots, which were harvested, weighed (typically 1 gfw), and placed in 500-mL mason jars equipped with a gas sampling port. Once the inoculated roots were sealed inside, 30 mL of acetylene gas (at STP) was withdrawn from the plastic pouch using a gas tight syringe and injected into the Mason jar through the inlet port. The jar was quickly vented to stabilize the inside pressure to 1 atmosphere and placed on a hot plate at 30 °C. Samples (0.1 mL) were removed at different incubation times for analysis of ethylene production using gas chromatography. Here, a Hewlett Packard 5890A gas chromatograph was equipped with a 2-m long, 1-mm inner diameter ShinCarbon ST packed column (Restek Corp., Bellefonte, PA, USA) and flame ionization detector. Column temperature was programmed as follows: the program started with a two-minute hold at 40 °C and then increased 10 °C min^−1^ to 250 °C. The injector temperature was maintained at 250 °C, and the flame ionization detector temperature was maintained at 300 °C. Chromatographic peaks for ethylene were measured using PeakSimple™ (v4.9) chromatography software (SRI Corp., Torrance, CA, USA) and quantified against ethylene standards. Ethylene was eluted at a retention time of 13 min.

### 2.9. Statistical Analysis

Data were subjected to the Shapiro–Wilk normality test to identify outliers, and thus all data groups reflected normal distributions. Data were analyzed using Student’s *t*-test for pairwise comparisons made between treatment types. Statistical significance was set at *p* < 0.05.

## 3. Results and Discussion

### 3.1. Microorganisms Can Influence Their Host’s Ability to Assimilate Carbon and Allocate It Belowground

**Figure 1:**
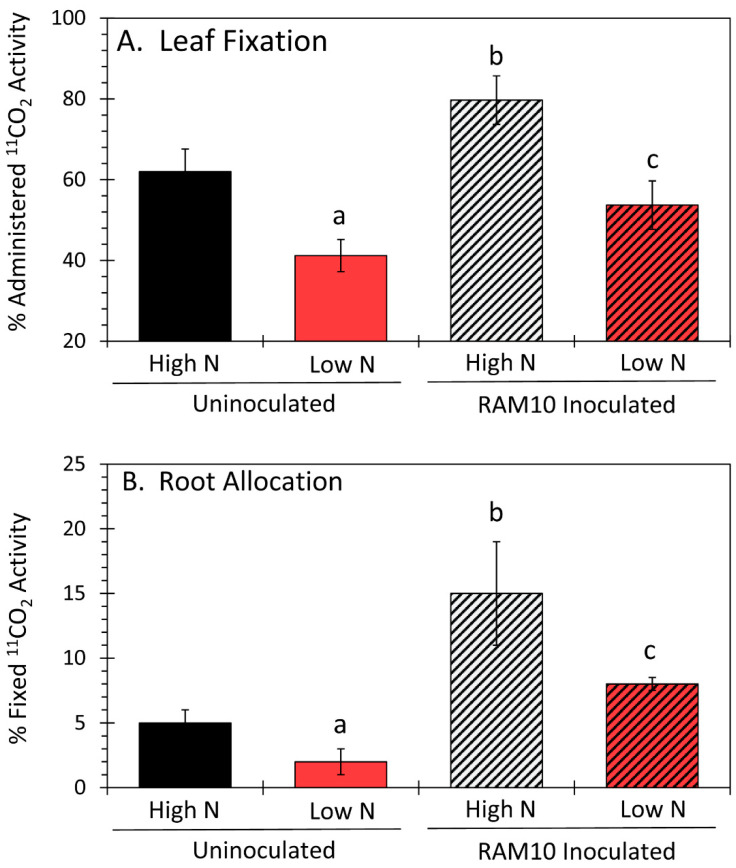
Levels of ^11^CO_2_ fixation are shown in (**A**) as a relative percentage of the administered ^11^CO_2_ pulse. All data were normalized to a standard fresh mass of leaf tissue. Levels of root allocation of ^11^C-photosynthates are presented in (**B**) as the percentage of the radioactivity fixed by the plant. Data reflect N = 6 plant replicates ± SE. Levels of significance (*p* < 0.05) were denoted by lowercase letters, where the letter “a” shows significance in data comparing uninoculated plants grown under low N to uninoculated plants grown under high N, the letter “b” shows significance in data comparing RAM10 inoculated plants grown under high N to uninoculated plants grown under the same conditions, and the letter “c” shows significance in data comparing RAM10 inoculated plants grown under low N to uninoculated plants grown under the same conditions.

To better understand how carbon resources derived from the host plant can influence microbial growth and their biological functions, such as BNF, we first examined how the status of N during plant growth affected carbon uptake and allocation of resources, then re-examined the influence of *H. seropedicae* inoculation on these plant processes. As expected, plants grown under N-limiting conditions exhibited significantly lower leaf fixation of ^11^CO_2_ (Figure 1A). This is because plants strive to balance their carbon uptake through leaf photosynthesis with that of the available nitrogen in the soil [22]. Reduction in carbon uptake also significantly reduced root allocation of ^11^C-photosynthates (Figure 1B). However, inoculation with RAM10 *H. serpedicae* under high-N growth conditions significantly increased ^11^CO_2_ fixation over that of uninoculated plants (Figure 1A), which boosted root allocation of carbon resources. Similar behavior was seen when plants were inoculated under low-N growth, although not to the same extent. The growth-promoting effects of *H. seropedicae* may or may not be due to BNF. As pointed out earlier, PGPB can boost plant fitness through several possible mechanisms.

### 3.2. Microorganisms Can Influence Their Host’s Carbon Metabolism

Much of the behavior described above for carbon resource allocation might be attributable to microbial influences on plant central carbon metabolism. Indeed, our past work using *A. brasilense* bacteria inoculants in maize demonstrated that host metabolism can be significantly altered by the presence of microorganisms growing on the roots [23]. This response can be beneficial to the microorganisms by providing them with carbon resources.

**Figure 2:**
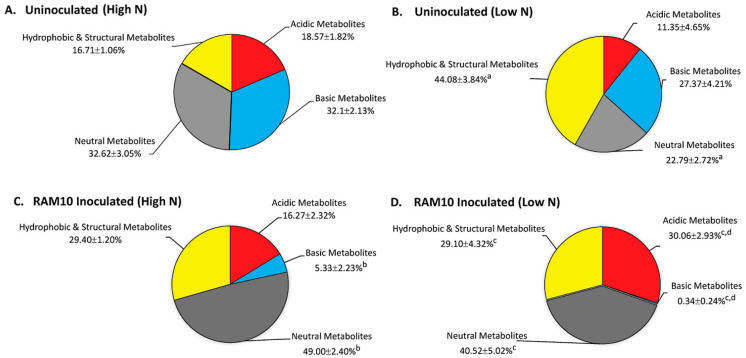
Carbon-11 aids in mapping changes in maize carbon metabolism as a function of N levels during plant growth and as a function of inoculation with RAM10 bacteria. The pie charts reflect the partitioning of “new” carbon (as ^11^C) into different metabolite pools of the load leaf tissue when harvested 20 min after the initial ^11^CO_2_ pulse. Data (mean ± SE) reflects N = 6–9 plant replicates. Pie sections notated with an “a” reflect statistical significance (*p* < 0.05) in a comparison between the uninoculated plants grown under low-N (**B**) and uninoculated plants grown under high-N (**A**) conditions. Pie sections denoted with a letter “b” reflect statistical significance (*p* < 0.05) in a comparison between RAM10-inoculated plants grown under high-N (**C**) and uninoculated plants grown under the same high-N conditions. Pie sections denoted with a letter “c” reflect statistical significance (*p* < 0.05) in a comparison between RAM10-inoculated plants grown under low-N (**D**) and uninoculated plants grown under the same low-N conditions. Pie sections denoted with a letter “d” reflect statistical significance (*p* < 0.05) in a comparison between RAM10-inoculated plants grown under low-N (**D**) conditions and inoculated plants grown under high-N conditions.

Using solvent extraction of plant leaf tissue coupled with ion exchange chromatography [23], we were able to examine how the host plant’s new carbon resources (as ^11^C) are being partitioned amongst the different pools of metabolites as a function of N limitation with and without microbial inoculation. An examination of results for uninoculated plants grown under high N (Figure 2A) vs. low N (Figure 2B) revealed significant increases in the hydrophobic and structural fraction under low N growth that was compensated by a significant reduction of the neutral metabolite fraction. Inoculation of plants with RAM10 *H. seropedicae* and grown under a high N regime (Figure 2C) caused a significant reduction in the basic metabolite fraction in support of a significant increase in the neutral metabolite fraction. Our past studies have revealed that this neutral metabolite fraction is comprised largely of soluble sugars [24]. Inoculation of plants with RAM10 *H. seropedicae* and grown under a low N regime caused similar behavior. The neutral metabolite fraction continued to remain significantly elevated while the basic metabolite fraction was nearly eliminated. The one difference noted between the two growth regimes is the presence of bacteria significantly elevated the acidic metabolite fraction. We note that *H. seropedicae* relies on acidic substrates such as malic acid as a carbon source for growth [7].

Altogether, our results suggest that *H. seropedicae* can manipulate its host’s carbon metabolism in a way that benefits microbial growth by generating more mobile plant substrates that can transport belowground to the roots. Indeed, this theory is supported by the responses of the host plant that were demonstrated in Figure 1 for ^11^CO_2_ fixation and transport of ^11^C-photosynthates belowground.

### 3.3. Effect of the N Environment on BNF and Its Influence on Microbial Demand for Plant-Borne Carbon

**Figure 3:**
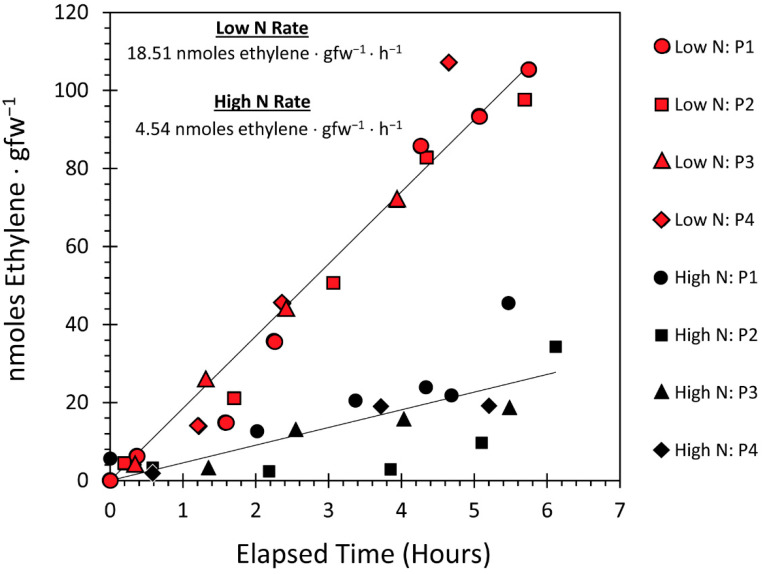
Results from acetyene reduction assays are shown in this figure for excised maize roots colonized by RAM10 *H. seropedicae*. The amount of ethylene produced (as nmole ethylene gfw^−1^ of root tissue in the sample) represents a measure of nitrogenase activity. Each root sample was analyzed repeatedly over the time course of 6 h from introduction of acetylene gas to the sample. Four biological replicates (labeled P1–P4) were performed for inoculated plants grown under low N (1 mM nitrate) and inoculated plants grown under high N (10 mM nitrate). Linear regression analysis was applied to arrive at the trendlines shown in the plot where slopes of these trendlines were calculated for the different N growth regimes, providing rates for nitrogenase activity in reducing acetylene-to-ethylene.

Using the acetylene reduction assay, we examined the influence of the N environment (present as nitrate) during plant growth on the functional behavior of root-colonizing *H. seropedicae* for fixing N_2_. Results summarized in Figure 3 as a plot of the ethylene production over time on exposure to acetylene gas revealed that nitrogenase activity was four times higher when inoculated plants were grown under the low-N regime than under the high-N regime. This result correlates well with prior observations where purified nitrogenase enzyme could be de-activated in the presence of extraneous ammonium or nitrate [10,11,12]. What is surprising here is while *H. seropedicae* prefers to colonize the inner root compartments rather than the outer root surface [7,18], this preferred mode of colonization did little to shield the bacteria from the surrounding N environment.

**Figure 4:**
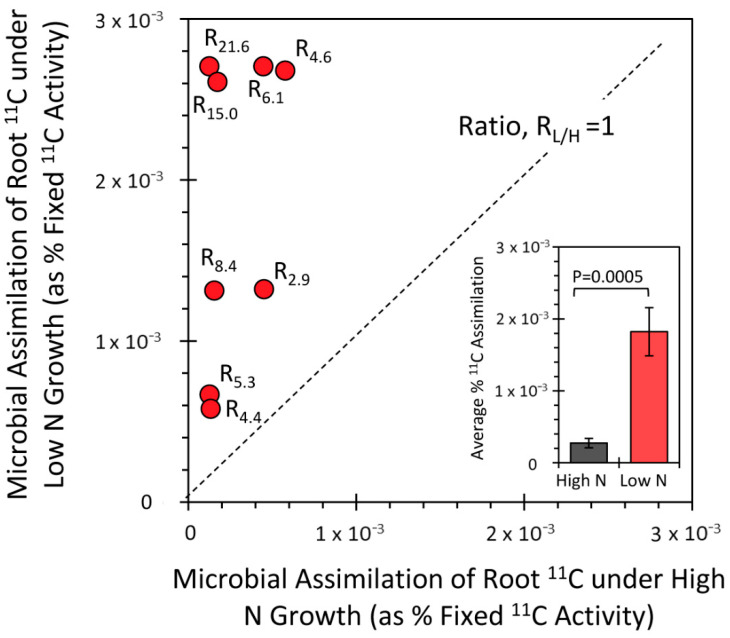
Results from the saline root sonication are presented in this figure as a scatter plot of microbial assimilation of ^11^C under low N growth (*y*-axis) versus microbial assimilation of ^11^C under high N (*x*-axis). All data are presented as percentage of ^11^CO_2_ fixed by the host plant and have been normalized for differences in root mass, and for differences in leaf fixation, root allocation of ^11^C-photosynthates and microbial content on the basis of their fluorescence signature [18]. The dotted line represents a trend of ratio, R_L/H_ = 1 for ^11^C assimilation under low N vs. high N. Actual values are shown for R_L/H_ next to each scatter point and in all cases exceeded a value of 1.0. The inset bar graph reflects a plot of the mean R values ± SE for these data and the level of significance in the data as determined by Student’s *t*-test.

In subsequent experiments, we examined the influence of the N environment on the microorganism’s “appetite” for carbon using a ^11^C-tracing technique we previously developed [18]. Results from this study are reported in Figure 4 as a scatter plot of microbial assimilation of root ^11^C when grown under the low N growth regime versus microbial assimilation of root ^11^C when grown under the high N regime. Because of differences in the host’s responses to N status relative to ^11^CO_2_ fixation and allocation of ^11^C-photosynthates to the roots, the data were normalized to standard levels of fixation and allocation. Further normalizations were applied to reflect differences in root mass and microbial content in that tissue that went into the analysis. One standard root mass with microbial content value (as measured by the GFP signature) was used to normalize all samples. These corrections had the effect of “leveling the playing field”, enabling us to directly assess microbial demand for carbon (i.e., their “appetite” for carbon) for the different N growth regimes. Altogether, the data presented here consistently showed ratios of microbial carbon demand greater than 1 (as shown by the ratio R_L/H_ reflecting microbial ^11^C assimilation when plants were grown under low-N vs. high-N regimes). This was consistent with our finding that under low N conditions, nitrogenase activity is greater. Thus, the energy demands of the microorganisms growing under these low N conditions must be higher, necessitating increased carbon assimilation.

### 3.4. Effect of the N Environment on Microbial Growth Performance

**Figure 5:**
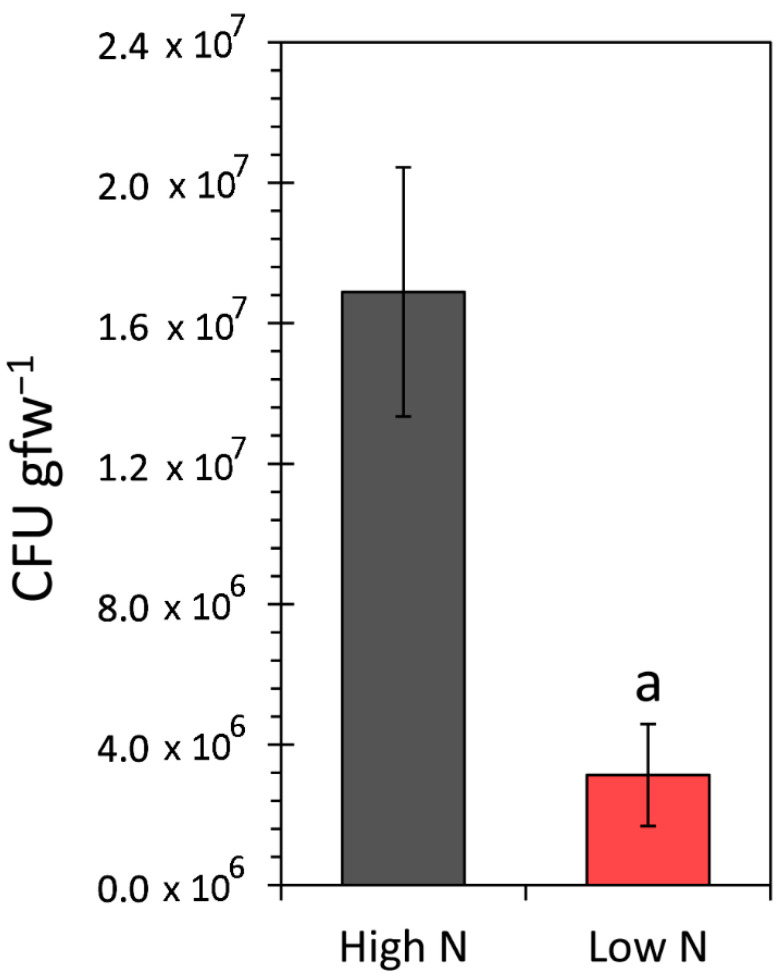
Results from microbial growth performance on live maize roots as determined by tissue drop plate assays are presented in this figure for growth regimes, including low N (1 mM nitrate) and high N (10 mM nitrate). Microbial content is presented as colony-forming units per gram of fresh root tissue (CFU gfw^−1^) subjected to the analysis. Data bars reflect means ± SE for N = 8 biological replicates under the high-N growth conditions and N = 7 replicates under the low-N conditions. Statistical significance (*p* < 0.05) is depicted by the letter “a”.

Drop plate assays were performed on root-colonized tissues [7] to determine the extent of their growth performance under low-N and high-N regimes. Results in Figure 5 show a significant reduction in microbial growth under low N. Even though the microbial “appetite” for carbon was high under these circumstances, less carbon was fixed by the host, causing less carbon to transport to the roots to satisfy the microbial carbon demands.

**Figure 6:**
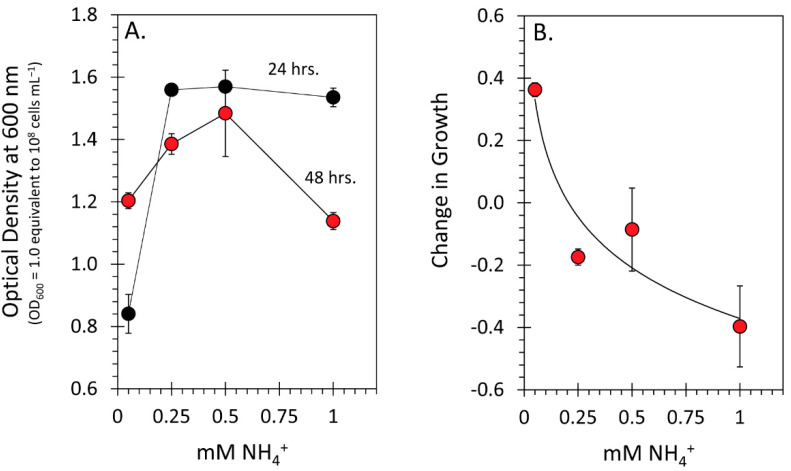
Results from microbial growth performance, as determined by the optical density measured at 600 nm, are presented in this figure for liquid cultures possessing different levels of extraneous ammonium chloride. Determinations of microbial growth were taken at 24 h and 48 h (**A**). The change in growth performance presented in (**B**) reflects the difference in optical densities between 48 h and 24 h values. Data in both panels reflect mean values ± SE for N = 3 biological replicates.

We also conducted in vitro bioassays examining microbial growth performance in liquid cultures possessing 0.05, 0.25, 0.50, and 1.0 mM NH_4_Cl. In all cases, microorganisms were exposed to an excess of carbon resources present as malic acid according to a prior growth protocol [7]. Here, we found that microbial growth increased rapidly from 0.05 to 0.25 mM ammonium and plateaued for the 24 h timepoints (Figure 6A). When measurements were taken at the 48 h timepoint, microbial growth appeared to peak at a 0.5 mM ammonia concentration, then declined at higher levels. A plot of the difference between the 48 h timepoint and the 24 h timepoint revealed an exponential falloff in microbial growth with increasing ammonium concentration. Independent of the host, microbial growth was higher under low N conditions than high N conditions when there was an ample supply of carbon resources.

## 4. Conclusions

To summarize, we found that the BNF capability of root-associating *H. seropedicae* is sensitive to extraneous nitrate decreasing in capacity at the higher N level. Furthermore, our ^11^C radiotracing studies revealed that the increased activity of nitrogenase in root-associating *H. seropedicae* grown under low N boosted their demand for carbon resources in support of the high energy demands of BNF. Unfortunately, we found that while the low-N regime was optimal for microbial function, their growth performance was significantly diminished due to the reduction in host carbon input and the reduction in allocation of carbon resources to the roots. Because our in vitro growth assay showed higher microbial growth under N-limiting conditions when the carbon supply was abundant, we suggest that a possible way to utilize these PGPB in the field under N-limiting conditions would be to provide a source of organic carbon to the soil. Such a practice would not have the same ecological consequences as fertilization with synthetic N, and likely would cost less to implement.
